# Effects of sea salt aerosols on precipitation and upper troposphere/lower stratosphere water vapour in tropical cyclone systems

**DOI:** 10.1038/s41598-019-51757-x

**Published:** 2019-10-22

**Authors:** Baolin Jiang, Dongdong Wang, Xiaodian Shen, Junwen Chen, Wenshi Lin

**Affiliations:** 1 School of Atmospheric Sciences, and Guangdong Province Key Laboratory for Climate Change and Natural Disaster Studies, Sun Yat-sen University, Guangzhou, Guangdong China; 2 Southern Marine Science and Engineering Guangdong Laboratory (Zhuhai), Zhuhai, China

**Keywords:** Atmospheric dynamics, Climate and Earth system modelling

## Abstract

The effects of sea salt aerosols (SSA) on cloud microphysical processes, precipitation, and upper troposphere/lower stratosphere water vapour in tropical cyclones were studied with the Weather Research and Forecasting with Chemistry model. Two numerical experiments were conducted: a control experiment (CTL) and an experiment with sea salt emission intensity one-tenth of that in the CTL experiment (CLEAN). Results show increased SSA concentrations, increased production rates of auto-conversion of cloud water to form rain, and increased accretion of cloud water by rain in the CTL experiment, leading to an increase in the precipitation amount. The peak value of precipitation is ~17 mm/h in the CTL experiment and ~13 mm/h in the CLEAN experiment, a difference of ~30%. The CTL experiment has more intense vertical movement in the eyewall and thus more water vapour is transported to the upper atmosphere, which promotes cloud ice deposition. This process consumes more water vapour, which makes the CTL experiment drier in the upper troposphere/lower stratosphere layer (altitude above 17 km). At 18–20 km altitude, the domain-averaged water vapour mixing ratio of the CTL experiment is ~0.02 ppmv lower than that of the CLEAN experiment. SSA have the effect of strengthening tropical cyclones and increasing precipitation.

## Introduction

Aerosols acting as cloud condensation nuclei (CCN) can affect cloud microphysical processes, the cloud water path, cloud lifetime, and cloud albedo^1^, but their effects are unclear^2^. The tropical cyclone (TC) is an important weather system that can be affected by aerosols. High anthropogenic aerosol concentrations increase the cloud water number concentration and mixing ratio, which suppresses the collision of cloud water droplets^3–8^. Higher numbers of cloud water droplets of a relatively small size at the TC periphery tend to enhance convection, lightning activity, and precipitation in the area^9–12^. Convective motion along the TC periphery may impede low-level moisture transport into the TC core and weaken the TC intensity^13,14^. Anthropogenic aerosols decreased the frequency of the North Atlantic TC over the twentieth century^15^. Moreover, rich anthropogenic aerosols may decrease TC intensity^16^.

The role of dust aerosols on TC systems has also been studied. Dust aerosols in the Saharan Desert are accompanied by stable, dry air, which leads to TC activity suppression over the Atlantic Ocean^17,18^. High dust aerosol concentrations over the northern Atlantic Ocean reduce the probability that tropical disturbances will develop into a TC^19^. Dust aerosols as CCN can change the cloud droplet number concentration and horizontal extent of the TC system^20^. Dust can lift cloud ice to high altitudes in a tropical convective system^21^.

Increased amounts of anthropogenic aerosols lead to more cloud water droplets of smaller size, which suppresses the formation of warm rain processes^5^. Aerosols activated as CCN may also influence cloud ice microphysical processes. When ice crystals are first formed, they grow mainly by cloud ice deposition, which consumes water vapour originating from either low-level lifted air or by the evaporation of cloud water^22^. This enhances the production of cloud ice, snow, and graupel, and cold rain is affected^23,24^. Specifically, cloud ice and ice-phase processes such as deposition and sublimation are important factors influencing the upper troposphere/lower stratosphere (UT/LS) water vapour content.

Water vapour is an important chemical component of the stratosphere and has an important influence on the global climate and the content of polar ozone. On the one hand, water vapour as a greenhouse gas can absorb infrared radiation, which has consequences for global climate change. On the other hand, it can be decomposed into hydroxyl radicals, which can alter the stratospheric ozone content. The two main sources of stratospheric water vapour are methane oxidation and convective transport from the troposphere to the stratosphere^25^. The tropics are the main source of global atmospheric water vapour, with stratospheric water vapour in tropical regions transmitted to the stratosphere in the middle and high latitudes by the Brewer–Dobson circulation^26^. An increase of 1% per year in stratospheric water vapour was observed during 1954–2000 based on ten datasets^27^. A tropical convection system brings water vapour into the stratospheric region, which can partially explain why the content of stratospheric water vapour has increased steadily for half a century^25^. The simulation of sea salt aerosols (SSA) with wide-size distribution with a bin microphysics model showed that cloud present continental properties in TC eyewall area^28^. Outside the eyewall, clouds remain maritime properties.

Convective activity occurs frequently in tropical areas, with some deep convection systems reaching several kilometres into the upper troposphere. The influence of convection systems on stratospheric water vapour is thought to generally occur through two mechanisms. The first mechanism is that the convection system dries the stratosphere. The upper troposphere is usually defined as the region of the troposphere with the lowest temperature, with the top of tropical troposphere usually located at an altitude from 14–18.5 km or 150–50 hPa^29^. When the clouds of convective systems reach the upper troposphere, the water vapour in the atmosphere condenses and falls because of the extremely low temperatures in this region. The stratosphere becomes dry and cool when penetrated by dehydrated air from the surrounding atmosphere^30–32^. The representative view is the “cold trap” hypothesis^31^.

The second mechanism is that cloud ice is injected into LS by convection systems and leads to the hydration of LS. Cirrus clouds have been observed in the tropical stratosphere^33,34^. The size distribution pattern of stratospheric cloud ice is very similar to that in the upper troposphere^35^, which implies that cloud ice may not completely fall to land after entering the troposphere, and may partly enter the upper troposphere or even the stratosphere. Therefore, the air may not be completely dehydrated in the troposphere. Recently, various observations and simulations have shown that convection systems can humidify the stratosphere. Water vapour transferred from the troposphere to the stratosphere is related to convection^33^. The convection system can reach the upper troposphere, while the cloud ice entering this layer sublimates and releases water vapour to humidify the UT/LS^36–40^.

Tropical cyclones are important convection systems in tropical areas. Although TCs only account for only 7% of convective systems in tropical areas. However, they account for 29% of tropical cloud in which the cloud top temperature is 15 K below tropopause temperature^41^. This implies that TCs play an important role in the change in stratospheric water vapour. Previous investigations have mainly focused on the influence of the deep convective activity on land^42–44^ or the change in CCN resulting from changes in cloud properties, which leads to alterations in the water vapour content in the UT/LS^30,45,46^. Satellite data were used to analyse the impact of TCs on the structure of the troposphere, as well as the ozone and water vapour contents over the northern Indian Ocean, which shows that the water vapour content increases in the lower stratosphere at distances 500–1000 km from the TC center^47^.

SSA, which have a broad size distribution and high hygroscopicity, can promote the formation of rain and convective precipitation^9,48^, which may influence the UT/LS water vapour content. Although the response of TC systems to anthropogenic aerosols or dust aerosols has been previously investigated^5,17^. The investigation of SSA effect on UT/LS has received little attention. Therefore, investigations into the complex mechanisms and impacts of SSA on TCs and UT/LS water vapour are required. This study aims to improve the understanding of the influence of SSA on precipitation and the UT/LS water vapour content in a TC system by focusing on the following questions: how do cloud microphysical processes respond to SSA? How does the precipitation change? How does the UT/LS water vapour change under the effect of SSA?

## Model Description and Experimental Design

We used the Weather Research and Forecasting with Chemistry (WRF-Chem) model version 3.5.1, which is a fully-compressible and non-hydrostatic Euler model, employing dry hydrostatic terrain-following pressure for the vertical coordinate, an Arakawa C-grid staggering^49^ for the horizontal grid, and a Runge–Kutta time integration scheme^50^. Model physics include the radiation, planetary boundary-layer physics, surface physics, cloud microphysics, and cumulus parameterisation schemes. The WRF-Chem^51^ model is a fully online forecasting model with various coupled chemical and physical processes, such as transportation, deposition, emission, gas-phase chemistry, aqueous chemistry, aerosol effects, photolysis, and radiation. Interactions between the aerosols and clouds are considered by the model. Aerosols can be activated as CCN to affect the cloud properties and cloud processes. An activation parameterisation of multiple aerosol types^52^ is also included in the model.

We employed two domains (D01 and the nested domain D02), with horizontal resolutions of 9 km and 3 km, time steps of 30 s and 10 s, and grid points of 336 × 256 and 424 × 406, respectively. The top pressure of the model is 20 hPa, and the number of vertical layers is 52 to ensure a vertical resolution of the free atmosphere of about 500 m. A high vertical resolution is beneficial for the simulation of cloud physical processes and thermodynamics. The model simulation time is 72 h, from 0000 UTC 20 August 2017 to 0000 UTC 23 August 2017. The National Centers for Environmental Prediction final global tropospheric analysis (NCEP-FNL 1° × 1°) dataset (http://rda.ucar.edu/datasets/ds083.2) is deployed for the initial and boundary conditions of the model.

The Morrison cloud scheme^53^, a two-moment scheme that considers the mixing ratio and number concentration of five hydrometeors (cloud water, rain, snow, graupel, and cloud ice) was adopted here. The Morrison scheme contains 42 types of cloud microphysical processes, including warm cloud microphysical processes (e.g., the auto-conversion of cloud water to form rain and the accretion of cloud water by rain) and cold cloud microphysical processes (e.g., cloud ice homogeneous freezing, heterogeneous freezing, cloud ice sublimation, and riming). Other physical parameterisations employed include the Yonsei University planetary boundary-layer scheme^54^, the National Centers for Environmental Prediction, Oregon State University, Air Force, the Hydrologic Research Lab’s land-surface module^55,56^, and the Rapid Radiative Transfer Model for Global Climate Models^57,58^ longwave/shortwave radiation scheme. The cumulus parameterisation was not employed because the two domains have a sufficiently high horizontal resolution. In addition, the aerosol driver module adopts the Modal Aerosol Dynamics Model for Europe/Secondary Organic Aerosol Model^59,60^ and the Regional Acid Deposition Model Version 2^61^.

The scheme for the SSA emission intensity is adopted in the model^62,63^, for which the sea salt emission flux is a function of the sea surface wind speed. In the WRF-Chem model, discharged SSA are treated as sodium chloride, with a particle size distribution described as two overlapping log-normal distributions for each mode^59^. The dry radius of SSA range from 0.1 to 10 microns in WRF-Chem model. The formulation of SSA emission intensity is as follows:

1 $$dF/dr=1.373{W}_{10}^{3.41}{r}^{-A}(1+0.057{r}^{3.45})\times {10}^{1.19\exp (-B)}$$

where *W*_10_ is the sea surface wind speed, *A* = 4.7 (1 + *Θ r*)^C^, *B* = (0.433 log *r*)/0.433, *C* = −0.017*r*^*−*1.44^, *r* is the SSA radius, and *Θ* is the particle shape parameter.

Discharged SSA may be activated as CCN after dry and wet deposition, which can change the cloud water number concentration and water vapour condensation, resulting in further changes to the cloud attributes, cloud microphysical processes, and precipitation. We only consider SSA emission in the simulations.

Two numerical experiments were designed to study the effect of SSA. In the first experiment (denoted the CTL experiment), we used the sea salt emission intensity proposed by Gong *et al*.^62,63^, while the emission intensity in the second (denoted CLEAN) experiment is only one-tenth that in the CTL experiment.

### Sea salt aerosol effects on precipitation

The No. 13 typhoon “Hato” was simulated, which occurred in the north-western Pacific in 2017, reached a super TC status, and landed in Guangdong, China at 2100 UTC on 23 August 2017. At 2100 UTC on 21 August 2017, the simulated TC centre entered the domain D02, so the analysis time period presented here is from 2100 UTC 21 August to 0000 UTC 23 August 2017. All presented analysis data were derived from the D02 region.

**Figure 1:**
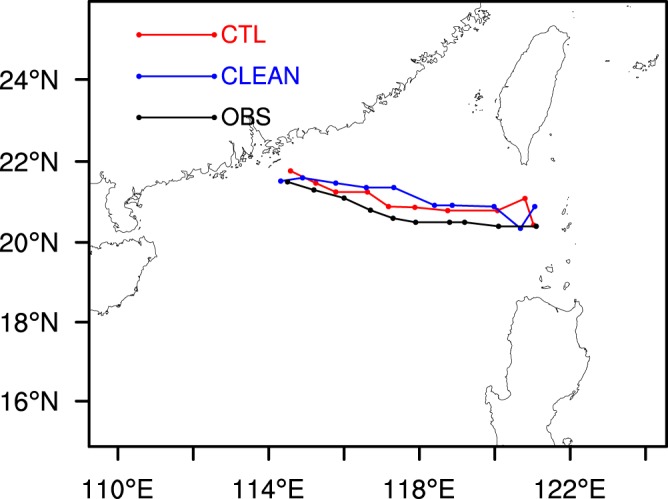
Simulated and observed 3-h interval tracks of TC “Hato”.

**Figure 2:**
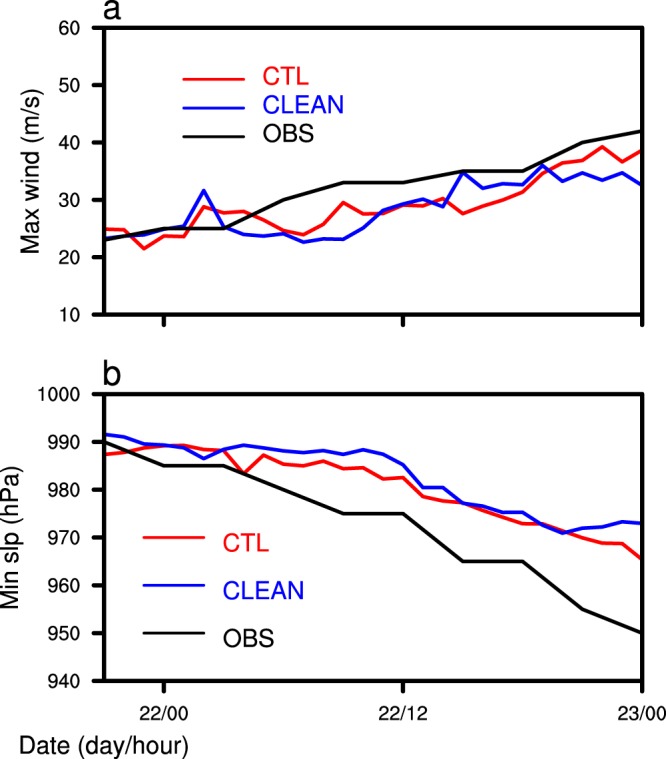
Temporal dependence of the maximum surface wind speed (**a**) and minimum sea level pressure (**b**).

Figure 1 shows the TC tracks of the simulations and observations and illustrates that TC Hato generally moved into the northwest, with the simulated tracks almost coincident with that observed, but slightly farther north. Figure 2 shows the TC intensity of simulations and observation and shows that the TC intensity is strengthened continuously during the simulation and observation period. The simulated maximum sea level wind speed is slightly less than that observed, whereas the simulated minimum sea level pressure is slightly higher than that observed. At 0000 UTC on 23 August, the simulated minimum sea level pressure is about 20 hPa higher than that observed. In general, the model simulates the tracks and intensities of the TC well compared with the observations. As there is no significant difference in these two aspects between the two simulations, this indicates the insensitivity of the TC track and intensity to the change in SSA concentration.

**Figure 3:**
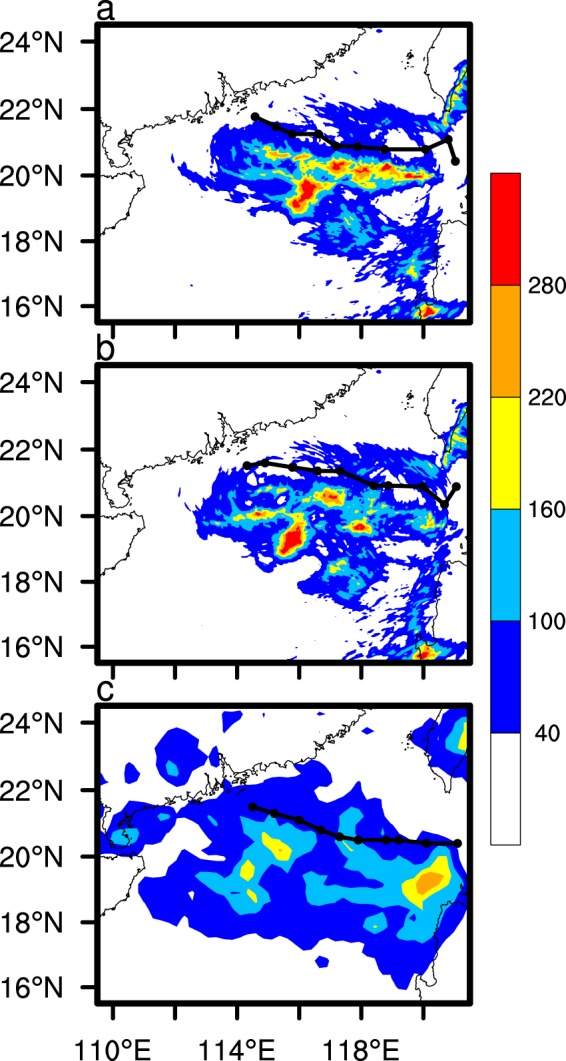
Accumulated precipitation (units: mm) of the CTL (**a**), and CLEAN simulations (**b**), and the TRMM satellite measurements (**c**). The simulated tracks of the two experiments and the observations are indicated by black lines. The time period is from 2100 UTC 21 August 2017 to 0000 UTC 23 August 2017.

Figure 3 presents the cumulative precipitation according to the simulations and the Tropical Rainfall Measuring Mission (TRMM) satellite data from 2100 UTC 21 August 2017 to 0000 UTC 23 August 2017. The horizontal resolution of the TRMM satellite cumulative precipitation data is 0.25°. The figure shows that the patterns of the simulated cumulative precipitation distribution are consistent with those observed, but slightly higher. The precipitation is generally distributed to the left side of the TC track. The cumulative precipitation of the CTL experiment in the eyewall area is significantly higher than that of the CLEAN experiment. Overall, the simulation results agree well with the observations, with the model simulating the TC track, intensity, and precipitation well.

**Figure 4:**
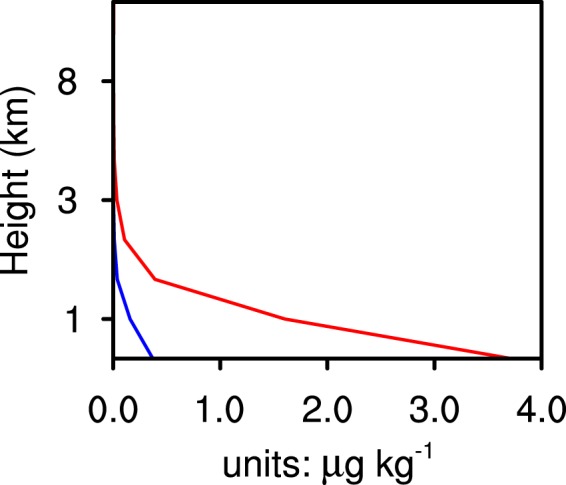
The average sea salt concentration. CTL simulation marked by red line; CLEAN simulation marked by blue line.

**Figure 5:**
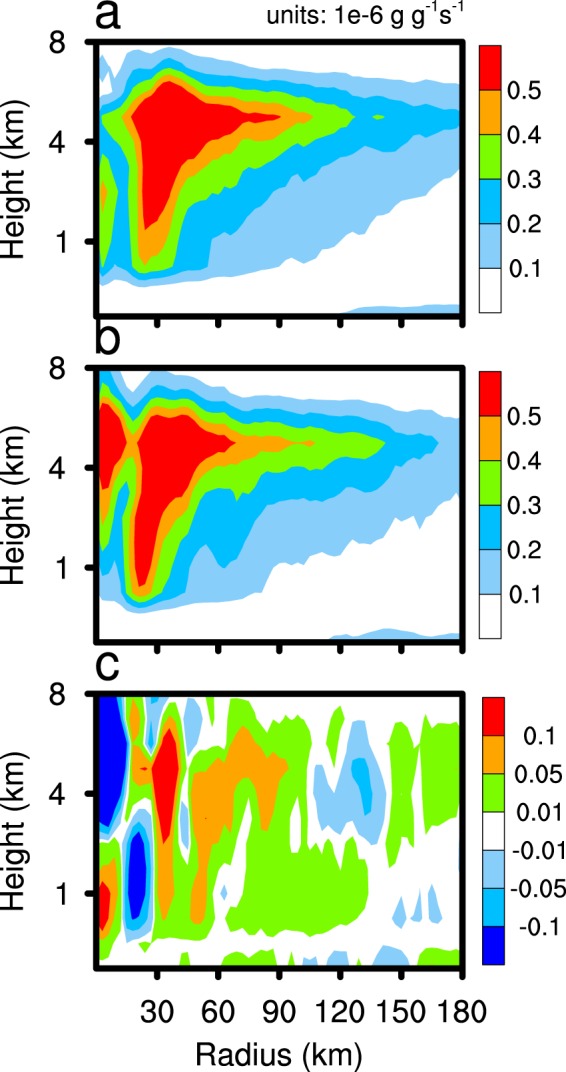
Time averaged from 2100 UTC 21 August 2017 to 0000 UTC 23 August 2017 and azimuthally averaged condensation production rates for the CTL simulation results (**a**), the CLEAN simulation (**b**), and CTL minus CLEAN (**c**).

**Figure 6:**
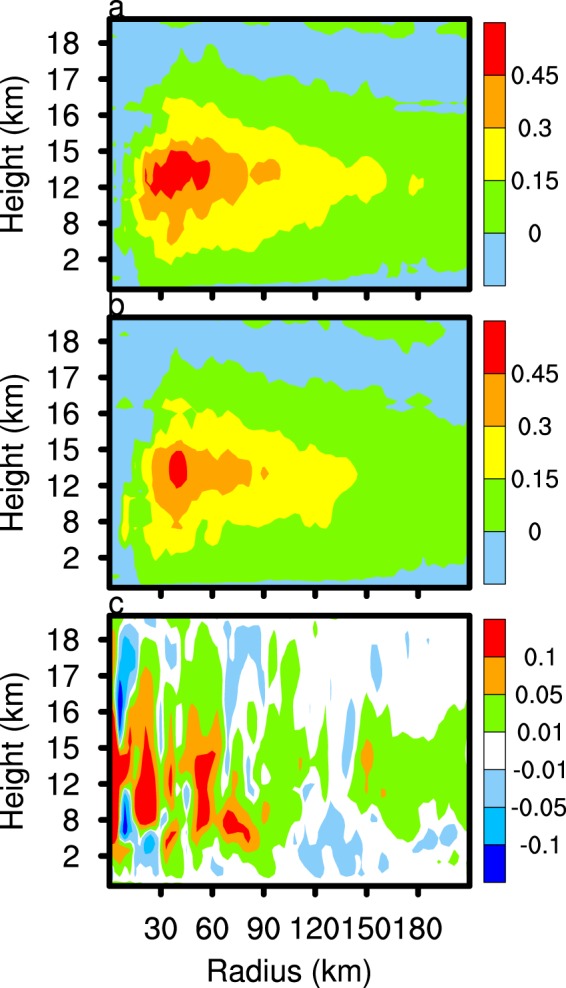
The time- and azimuthally-averaged vertical velocity component of the CTL simulations (**a**), the CLEAN simulations (**b**), and the CTL minus CLEAN simulations (**c**).

**Figure 7:**
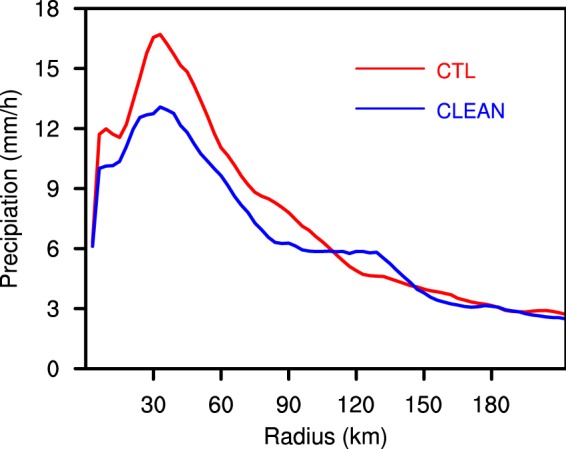
Time- and azimuthally-averaged precipitation of the CTL (red line) and CLEAN simulations (blue line) for the time period 2100 UTC 21 August 2017 to 0000 UTC 23 August 2017.

As the sea salt emission intensity of the CLEAN experiment is only one-tenth that of the CTL experiment, there is a clear difference in the SSA concentration between the two experiments. Figure 4 shows the SSA concentration vertical distribution of the two experiments. SSA are treated as CCN in the WRF-Chem model. The increase in SSA concentration is conducive to the increase in condensation. Figure 5 shows that the TC condensation process occurs mainly below 6 km within about 50 km radius, with the production rate of TC condensation reaching above 0.5 × 10^−6^ g g^−1^ s^−1^. Supersaturation is prognosticated in WRF-CHEM with the Morrison scheme. Supersaturation is affected by temperature and water vapour mixing ratio. Supersaturation of the CLEAN simulation is slightly larger than that in the CTL simulation at 1 km altitude (figure not shown), because the enhancement of condensation consumes more water vapour in the cloud base. The condensation production rate of the CTL experiment is higher than that of the CLEAN experiment, especially in the eyewall area, which releases a large amount of latent heat, thus enhancing vertical motion. Figure 6 reveals the difference of the vertical velocity component between the CTL and CLEAN experiments and illustrates a greater value for the CTL experiment than the CLEAN experiment in the eyewall area. The increase of the vertical velocity component promotes the development of convective motion, thereby increasing the precipitation in the TC eyewall area. This is consistent with Fig. 3, which shows the cumulative precipitation of the CTL experiment in the eyewall area to be significantly greater than that of the CLEAN experiment. Figure 7 demonstrates the radial distribution of average precipitation rate of the simulated TC. The peak precipitation of the simulated TC Hato is located 30–60 km from the TC centre, with the peak value of precipitation ≈17 mm/h in the CTL experiment and ≈13 mm/h in the CLEAN experiment, a value nearly 30% greater in the CTL with respect to the CLEAN experiment.

**Figure 8:**
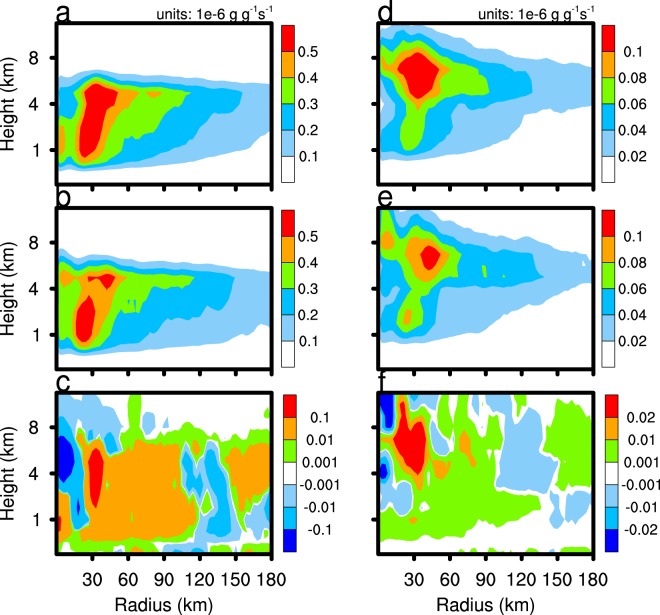
As in Fig. 5, but for the auto-conversion of cloud water to rain (left column) and the accretion of cloud water by rain (right column).

**Figure 9:**
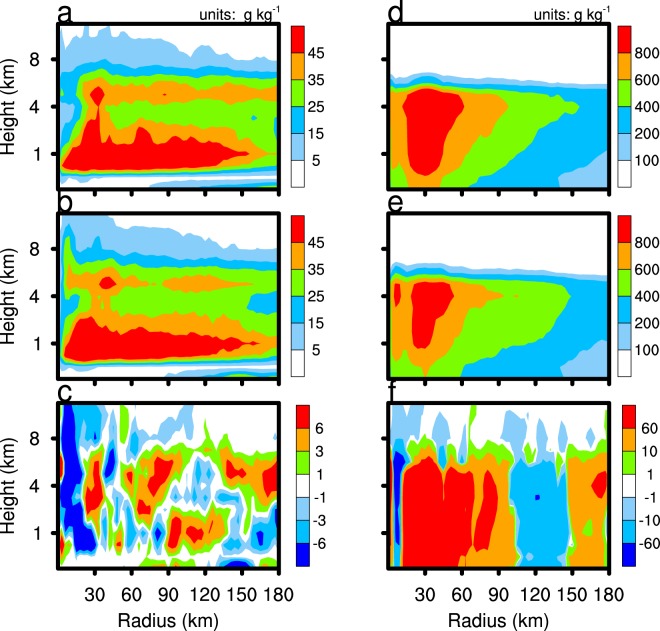
As in Fig. 5, but for the cloud water mixing ratio (left column) and rain mixing ratio (right column).

The two main microphysical processes that affect TC precipitation are auto-conversion of cloud water to form rain and the accretion of cloud water by rain, and both processes are stronger in the CTL experiment than the CLEAN experiment (Fig. 8). It is generally understood that an increase in anthropogenic aerosols can enhance CCN, which increases the competition for moisture between cloud water particles, and decreases the effective radius, so that collisions between cloud water droplets are reduced. However, as sea salt is a highly hygroscopic aerosol, the excessive humidity in TC systems leads to the rapid growth of SSA because of moisture absorption and generates a larger radius of cloud water. Precipitation enhancement results from increased production of cloud water in the CTL simulation. The accretion of cloud water by rain and the auto-conversion rate of cloud water of the CTL experiment exceeds that of the CLEAN experiment because the conditions are more favourable to the increase in cloud water number concentration and mixing ratio in the CTL experiment. As these two processes consume cloud water and produce rainwater, their enhancement increases the precipitation of the eyewall area and mixing ratio of rainwater in the CTL experiment (Fig. 9). The average rain mixing ratio of the CTL and CLEAN experiments is 106.8 and 97.4 g kg^−1^, respectively, and number concentrations is 14.3 × 10^3^ and 14.4 × 10^3^, respectively.

### Effects on water vapour in UT/LS

**Figure 10:**
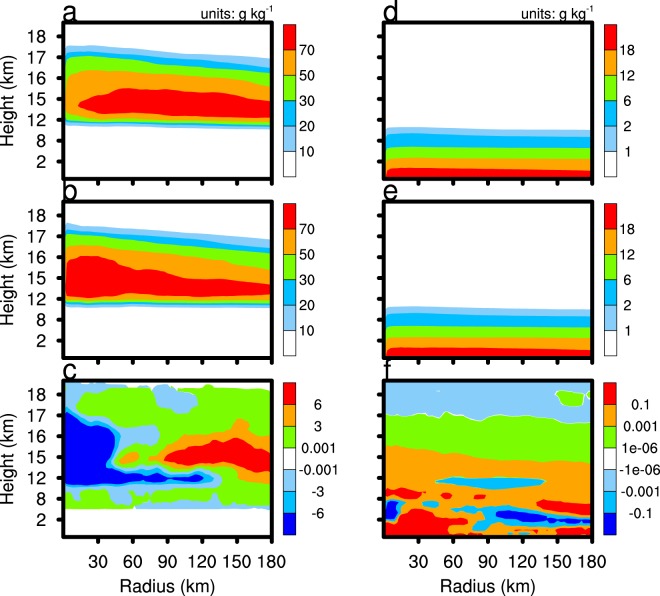
As in Fig. 5, but for the cloud ice mixing ratio (left column) and water vapour mixing ratio (right column).

The enhancement of vertical motion in the CTL experiment not only strengthens convective activity and precipitation in the experiment, but also transports more water vapour to the upper atmosphere in the TC system. Figure 10 indicates that, with the exception at the low-level periphery of the TC, the water vapour mixing ratio of the CTL experiment exceeds that of the CLEAN experiment below an altitude of 17 km, but the situation is reversed above 17 km in the upper troposphere. According to the mass conservation equation, the vertical velocity component of the eyewall area increases along with the flow toward the TC eyewall, which increases the transport of water vapour from the periphery of the TC to the eyewall area, and to higher levels.

**Figure 11:**
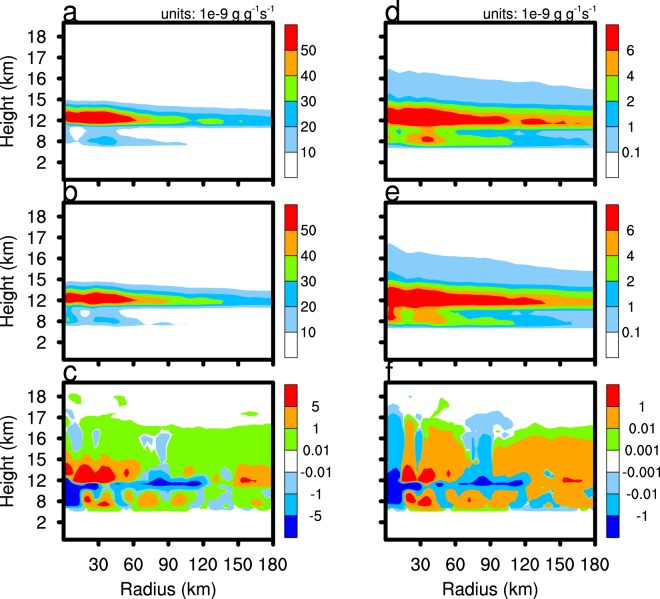
As in Fig. 5, but for the cloud ice deposition growth (left column) and cloud ice collisions (right column).

Any change in the amount of water vapour in the upper area of the TC will change the cloud ice content and the ice-phase microphysical processes. Cloud ice embryo formation is mainly the result of homogenous and heterogeneous freezing, and rime splintering. Once initiated, cloud ice deposition is the main microphysical process governing growth. Figure 10 reveals that the mixing ratio of cloud ice in the TC eyewall and periphery in the CTL experiment significantly exceeds that of the CLEAN experiment. In the TC centre, the mixing ratio of cloud ice decreases with the increase of the downdraft in the CTL experiment. Following the enhancement of water vapour mixing ratio below 17 km in the CTL experiment, the production rate of cloud ice deposition growth strengthens (Fig. 11), which increases the cloud ice mixing ratio.

**Figure 12:**
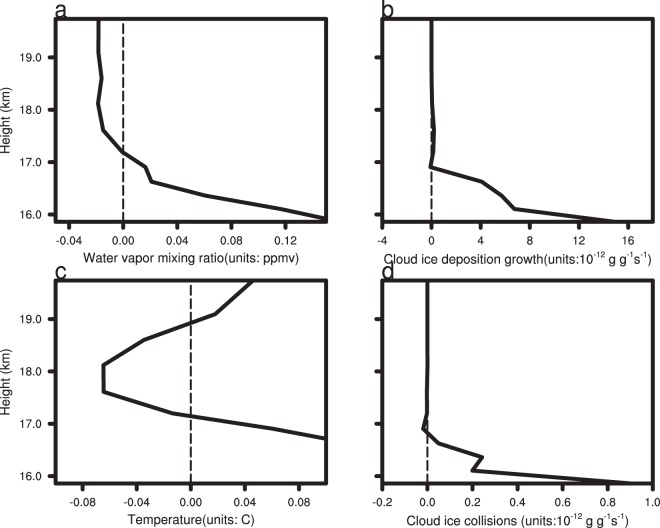
The domain D02 averaged and time averaged water vapour volume mixing ratio (**a**), cloud ice deposition growth profile (**b**), temperature (**c**), and cloud ice collisions (**d**) of the CTL minus CLEAN simulations. The time period is from 2100 UTC 21 August 2017 to 0000 UTC 23 August 2017.

The content of water vapour and cloud ice in the upper troposphere has an important influence on lower stratosphere moisture (Fig. 12). The area with the lowest atmospheric temperature (about 17.2 km, figure not shown) is usually defined as the tropopause. Figure 12c shows that the tropopause temperature of CTL is slightly lower than that of CLEAN simulation. Figure 12a shows the domain D02 averaged and time-averaged water vapour profile difference of the CTL and CLEAN experiments (CTL minus CLEAN). The water vapour mixing ratio of the CTL experiment exceeds that of the CLEAN experiment below 17.2 km, with the situation reversed above 17.2 km. At the altitude of 18–20 km, the water vapour mixing ratio in the CTL experiment is about 0.02 ppmv lower than the CLEAN experiment. In the troposphere below 17 km, the cloud ice deposition growth rate of the TC in the CTL experiment exceeds that of the CLEAN experiment, so the former consumes more water vapour within the tropopause, which dries the lower stratosphere. The average number concentration of cloud ice in the CTL and CLEAN experiments is 4.9 × 10^5^ and 4.8 × 10^5^, respectively. The increase of cloud ice mixing ratio and number concentration in the CTL experiment is advantageous to ice microphysical processes, such as cloud ice collisions (Figs 11, 12d), cloud ice collected by snow, and graupel formation. These cloud microphysical processes consume cloud ice and may enhance ice particle sedimentation. More attention, the lack of the process of ice nuclei activated by SSA in the Morrison scheme allows for the possibility of deviations from our results in real word. Overall, the enhancement of cloud ice deposition growth, which consumes more water vapour in the upper troposphere in the CTL experiment compared with the CLEAN experiment, enhances air drying.

## Summary and Discussion

We studied the effect of SSA on cloud microphysical processes and the precipitation of the TC Hato using the WRF-Chem model, with the focus on the influence of SSA on precipitation and the water vapour of the UT/LS layer. The sea salt emission is based on a parameterised scheme. Two simulation experiments denoted as CTL and CLEAN are so defined according to their sea salt emission intensity. They demonstrate that the WRF-Chem model simulates the TC track, intensity, and precipitation well. However, the effect of a different sea salt emission intensity on the TC track and intensity is not obvious. As a highly hygroscopic aerosol, SSA can be activated as CCN to influence the cloud microphysical processes and precipitation of the TC. SSA can also absorb moisture and generate large droplets of cloud water, which enhances the collision efficiency of cloud water. The increase of SSA concentration is conducive to the formation of cloud water, which promotes the accretion of cloud water by rain. Therefore, the auto-conversion of cloud water to form rain and the accretion of cloud water by rain in the CTL experiment are stronger processes than in the CLEAN experiment. The increase of these two cloud microphysical processes results in a larger rain mixing ratio and higher precipitation in the CTL experiment. The peak value of the radial distribution of precipitation in the CTL and CLEAN experiments is 17 and 13 mm/h, respectively, which is an approximately 30% difference considering the order of magnitude difference in sea salt emission intensity.

The increase of SSA in the CTL experiment leads to the enhancement of condensation. The latent heat released by vapour condensation promotes the development of convective movement in the TC eyewall, so that more water vapour is transported to the upper layers of the TC, which promotes cloud ice deposition growth in the CTL experiment. Therefore, the cloud ice mixing ratio and number concentration in the CTL experiment are increased slightly compared with those in the CLEAN experiment. Overall, SSA leads to more water vapour condensation, increased eyewall vertical velocity, cloud water content, and cloud ice content, which is similar to the findings of previous research^28^. In the CTL experiment, the growth of cloud ice deposition consumes more water vapour in the upper troposphere area, which dries the air and decreases the water vapour mixing ratio of the lower stratosphere. This conclusion is consistent with the “cold trap” hypothesis proposed^31^.

It is worth noting that the formula proposed based on global sea salt emission observations needs observational support during a TC system because of the difficulty in recording quality data under such conditions. We studied the impact of SSA on TC precipitation and UT/LS water vapour, but the effect of aerosols as ice nuclei was not included because the Morrison scheme only considers the heterogeneous freezing process. In the future, we will further study the role of SSA as ice nuclei.
